# Genome-wide autosomal, mtDNA, and Y chromosome analysis of King Bela III of the Hungarian Arpad dynasty

**DOI:** 10.1038/s41598-021-98796-x

**Published:** 2021-09-28

**Authors:** Chuan-Chao Wang, Cosimo Posth, Anja Furtwängler, Katalin Sümegi, Zsolt Bánfai, Miklós Kásler, Johannes Krause, Béla Melegh

**Affiliations:** 1 Department of Archaeogenetics, Max Planck Institute for the Science of Human History, 07745 Jena, Germany; 2 Department of Anthropology and Ethnology, Institute of Anthropology, School of Sociology and Anthropology, State Key Laboratory of Cellular Stress Biology, State Key Laboratory of Marine Environmental Science, National Institute for Data Science in Health and Medicine, School of Life Sciences, Xiamen University, Xiamen, 361005 China; 3 Institute for Archaeological Sciences, Archaeo- and Palaeogenetics, University of Tübingen, 72070 Tübingen, Germany; 4 Department of Medical Genetics, Medical School, University of Pécs, Szigeti u. 12, Pécs, 7624 Hungary; 5 Szentágothai Research Center, University of Pécs, Ifjúság út 24, Pécs, 7624 Hungary; 6 Department of Biochemistry and Medical Chemistry, Medical School, University of Pécs, Szigeti u. 12, Pécs, 7624 Hungary; 7 National Institute of Oncology, Rácz Gy. u. 7-9, Budapest, 1122 Hungary

**Keywords:** Evolutionary biology, Genetic markers, Genotype, Haplotypes, Medical genetics, Population genetics

## Abstract

The ancient Hungarians, “Madzsars”, established their control of the Carpathian Basin in the late ninth century and founded the Hungarian Kingdom around 1000AD. The origin of the Magyars as a tribal federation has been much debated in the past. From the time of the conquest to the early fourteenth century they were ruled by descendants of the Arpad family. In order to learn more about the genetic origin of this family, we here analyzed the genome of Bela III one of the most prominent members of the early Hungarian dynasty that ruled the Hungarian Kingdom from 1172 to 1196. The Y-Chromosome of Bela III belongs to haplogroup R1a-Z2123 that is today found in highest frequency in Central Asia, supporting a Central Asian origin for the ruling lineage of the Hungarian kingdom. The autosomal DNA profile of Bela III, however, falls within the genetic variation of present-day east European populations. This is further supported through his mtDNA genome that belongs to haplogroup H, the most common European maternal lineage, but also found in Central Asia. However, we didn’t find an exact haplotype match for Bela III. The typical autosomal and maternal Central Eastern European ancestry among Bela III autosomes might be best explained by consecutive intermarriage with local European ruling families.

## Introduction

The vast majority of Europeans today speak Indo-European languages, but their origins and dispersals are highly debated topics. Bayesian methods applied to date the root of Indo-European languages provide an age estimate of ~ 6,000 years before Common Era (BCE) and suggest Anatolia as the homeland of Proto-Indo-European^1,2^. An alternative hypothesis proposes that Proto-Indo-European speakers were nomadic pastoralists of the Pontic–Caspian steppe and their languages spread into Europe after the invention of wheeled vehicles^3,4^. Genetic data from ancient human remains across Neolithic and Bronze Age Europe revealed a massive population turnover mediated by the expansion of pastoralists from the eastern European steppe starting around 3000 years before the common era (BCE)^5,6^. The described population movement might have been responsible for replacing the original languages and contributing the “steppe” genetic component that, together with “Hunter-Gatherer” and “Farming” ancestries, describe the genetic makeup of most Europeans today. From this steppe-Indo-European correlation, there are known exceptions like the Basques who despite carrying steppe ancestry speak a non-Indo-European language. Genetic studies have revealed that Basques are the closest modern-day population to Neolithic and Iron Age individuals from Iberia^7,8^ suggesting a possible survival of the local language from the Neolithic onwards. The second most spoken language family in Europe is the so-called Finno-Ugric (according to the theory of the language family-tree) that today is distributed across Finland, Estonia, western Russia and Hungary. This is divided into Finnic and Hungarian (also called Magyar), both part of the larger Uralic language branch, which is widespread as far as northeastern Eurasia^9^.

Genetic studies on modern-day populations have shown that in Europe genetic distance among populations has a high correlation to their geographical distance^10^. This, however, is not the case for present-day Finnish individuals that are displaced from the European genetic cluster towards East Eurasian populations. A recent ancient DNA study of Fenno-Scandian human remains identified an additional genetic contribution ultimately related to a Northeast Asian population (Nganasan) reaching this region by at least 3,500 BCE^11^. This genetic component is present in lower proportions in present-day Saami individuals from northern Finland and to an even lesser extent in Finns, who are largely admixed with central European ancestry. Notably, the distribution of the putative Siberian-related ancestry is present in most Uralic speaking populations but absent in modern-day Hungarians^12^. Contrary to Finns, Hungarians fall almost entirely within the genetic diversity of modern-day Europeans harboring only a minor East Asian component^13^. Genomic analyses of ancient human remains from Hungary have revealed a process of large genetic replacement during the Early Neolithic with the arrival of “Farming” ancestry followed by the resurgence of local “Hunter-Gatherer” ancestry in the Middle Neolithic, as observed in other European regions^14,15^. Furthermore, during the Late Neolithic-Early Bronze Age transition the “steppe” ancestry spread throughout Hungary providing the third genetic component present in most Europeans today^16^.

The following period of the genetic history in Hungary is less well characterized. In fact, almost all ancient DNA studies on post-Iron Age individuals have been relying on the genetic typing or sequencing of polymorphisms within the Y-chromosome (Ychr), on partial or complete mitochondrial DNA (mtDNA) sequences and on phenotypic SNPs^17–24^, which has not permitted a detailed genome-wide characterization of populations in Hungary through time. After the Iron Age from 35 BC to the early ninth century AD, Hungary was part of the Roman Empire and later underwent several “barbaric migrations” such as from the Huns between the 4th and the sixth century AD and from the Longobards in the sixth century AD, followed by the Avars between the late sixth century and the early ninth century AD^25^. Genomic data is so far available only from a Longobard-associated cemetery in Pannonia that revealed genetically heterogeneous individuals, thus suggesting that this group was a congregation of people with different origins not resembling previous or present-day Hungarian populations^26^.

According to available written records ca. 530 AD, King “Muageris”–which has been associated with the name “Magyar”–was the ruler of the Kutrigur Huns north of the Black Sea^27^. Centuries later Álmos the first, Great Prince of the Hungarians, organised the monarchic state in the same region ca. 850 AD, although the links with the previous populations are not fully deciphered. A few decades after the collapse of the Avar Khaganate (c. 822 AD), Álmos and his son Árpád conquered the Carpathian Basin (c. 862–895 AD)^28^. During the conquest, it is suggested that Hungarian conquerers, together with Turkic-speaking Kabars, assimilated Avars and Slavonic groups^29–31^.

Interestingly, the reconstructed genealogy of the so-called Árpád Dynasty (this term was initiated in the eighteenth century for the first royal house of Hungary; then the scholars named the dynasty after Álmos’ son Árpád who completed the Hungarian Conquest) (Figure S1)^32^ shows that a paternal line of inheritance has always been followed from Great Prince Álmos—who lead the initial conquest after/ca. 862 AD—to King Andrew III of Hungary, who died in 1301 AD, signing the dynasty’s end. Along this succession line, one of the most prominent kings was Bela III (1172–1196), the first king who adopted the "double cross" as the symbol of the Kingdom of Hungary (Figure S2). He was the son of King Geza II, married with Anna of Antioch from France and their first son later became King Emeric. Bela III was initially buried together with his wife Anna and possibly other unidentified members of the Árpád Dynasty in the Royal Basilica of Székesfehérvár but was later reburied in the Matthias church in Budapest. In 2012 as part of the exhumation of skeletons associated with the royal family, anatomical elements from King Bela III and Anna of Antioch were collected. Olasz et al. genotyped Y-STR haplotype of King Béla III and predicted he belonged to haplogroup (hg) R1a^33^. Nagy et al. reported the Y chromosome sequence of Bela III and found the lineage traced to the region centering near Northern Afghanistan about 4500 years ago and the present-day Bashkirs were his closest paternal kin with a separation date about 2000 years ago^34^. Here, we attempted a genome-wide characterization of the remains of Bela III in order to address whether the genome of King Bela III groups within the gene pool of central Asians or present-day Hungarians.

## Results

### Next generation sequencing

We collected four bone fragments from the tomb associated to King Bela III. DNA was extracted^35^ from those specimens and converted into single and double-stranded libraries (Table S1)^36,37^. After shallow shotgun screening, we further enriched six libraries for a targeted set of ~ 390 thousand or ~ 1.24 million SNPs across the human genome^38,39^ and sequenced on Illumina platforms (HiSeq4000, NextSeq500) (Table S2). The combined coverage on the targeted SNPs is 6.154X (1,090,066 SNPs).

All libraries showed the typical pattern of ancient DNA that is an increased C to T substitution rate towards the molecule ends (Table S2)^40^. Sex determination was performed inspecting the coverage of the captured SNPs overlapping sex chromosomes compared to the autosomes^41^. Bela III was assigned to be male (Table S3). All libraries belonging to Bela III consistently reported values of X-chromosome contamination^42^ below 2.5% as well as high DNA damage rates confirming the authenticity of the isolated DNA (Table S4).

### Mitochondrial DNA and Y chromosomal analysis

We performed mtDNA capture on Bela’s libraries and estimated mtDNA contamination level at 1% and determined his mtDNA hg (Table S5)^43^. Bela III carried hg H1b belonging to the mtDNA H clade that is the most common hg present in Europe today^44^. We found a list of polymorphisms that Bela III had against the Revised Cambridge Reference Sequence (rCRS)^45^: 263G, 315 + C, 750G, 1438G, 3010A, 3106A, 4769G, 8860G, 15326G, 16183C, 16189C, 16356C, 16519C. Our mtDNA result is consistent with the published mtDNA control region data as reported in Olasz et al.^33^. We used EMPOP (https://empop.online/) to search if this mitogenome haplotype has been reported before and where. There are 211 samples of H1b haplogroup mainly reported in Europe but also found in Central Asia in EMPOP, but we didn’t find an exact matched haplotype with that of Bela III.

The Y chromosomal mutations were inspected at all ~ 40,000 positions present in our captured SNP panel. We could identify 30 derived mutations stemming from the hg R node and leading to hg R1a1a1b2a-Z95 plus an additional mutation on a downstream SNP, which defines Y chromosomal hg R1a1a1b2a2a-Z2123 (Table S6). This is a sub-hg of R1a1a1b2-Z93, the main Asian branch of R1a that has particularly high frequencies in Central Asia^46^, which is consistent with the result reported in previous studies^33,34^.

### Genome-wide autosomal marker data analysis

**Figure 1:**
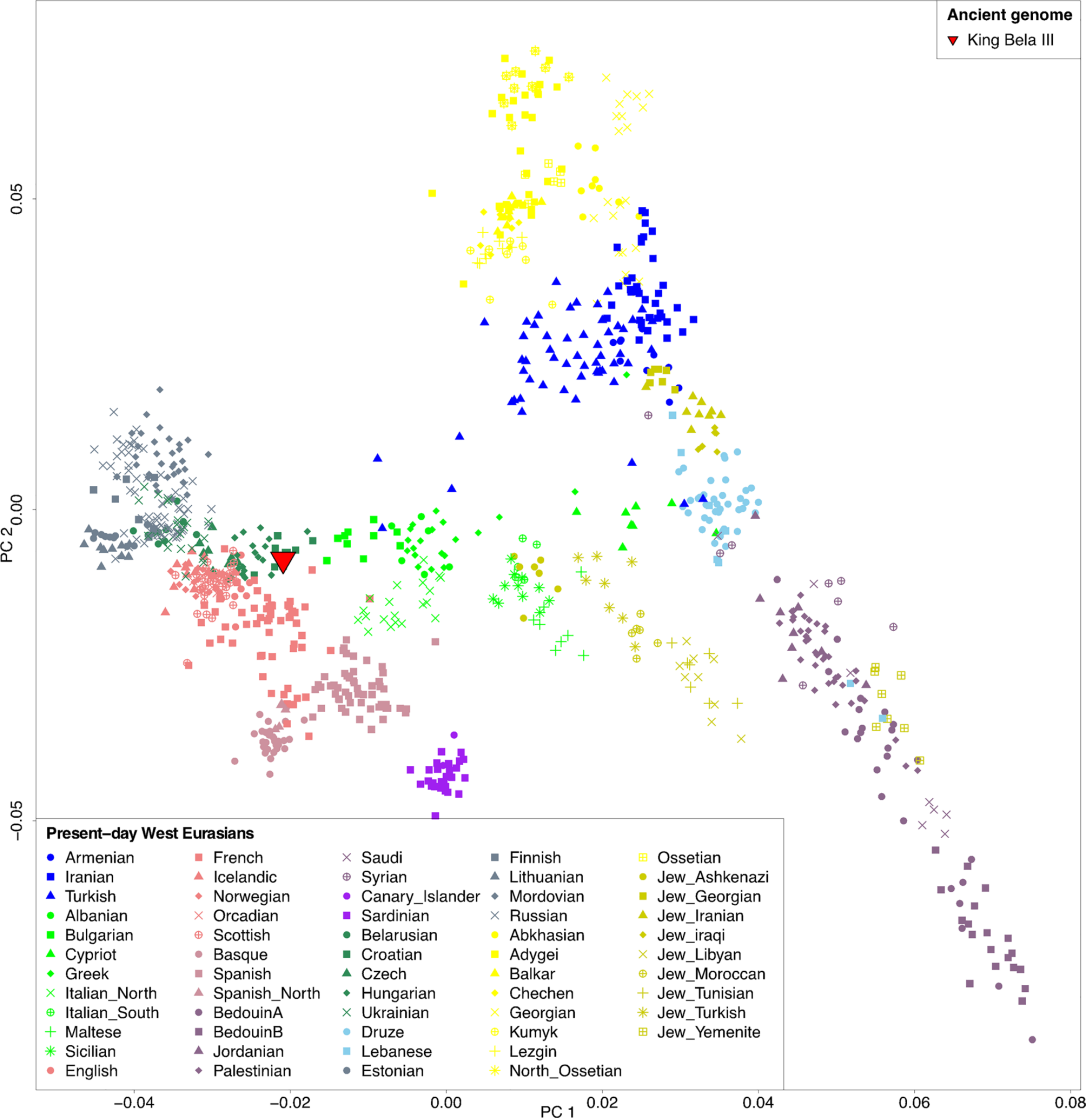
Principal component analysis of present-day west-Eurasians with the genome of Bela III projected.

We further investigated the ancestry of Bela III making use of the genome-wide captured data. We first projected the autosomal data onto the first two principal components (PCs)^47^ built with modern-day West-Eurasian genetic variation (Fig. 1) ^39^. The King’s genome falls in close proximity to present-day populations from Croatia and Hungary. Comparable patterns were obtained from the clustering algorithm implemented in ADMIXTURE^48^ where Bela III shares a similar genetic profile with present-day Eastern European populations (Figure S3).

**Figure 2:**
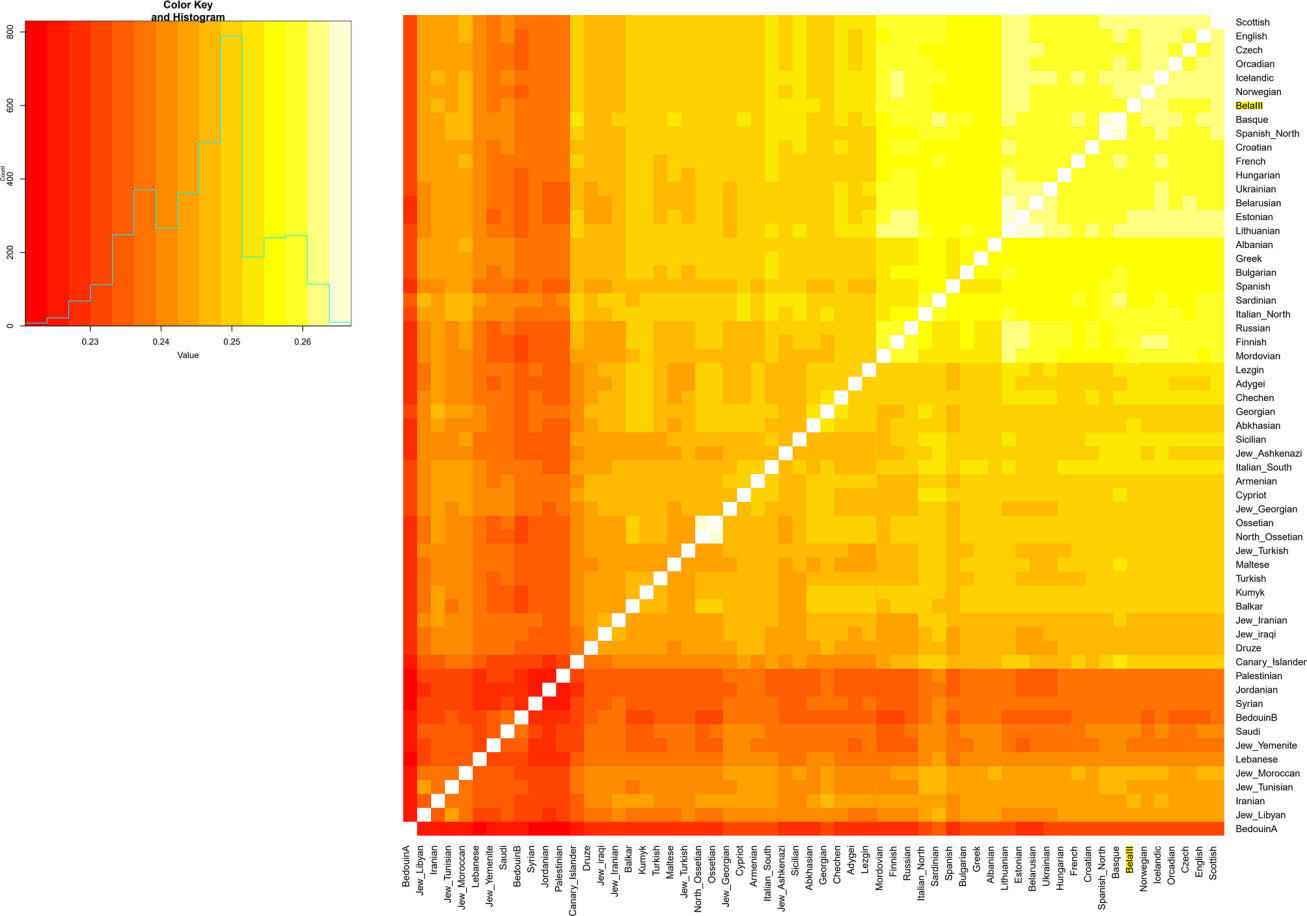
Pairwise *f*_*3*_-outgroup statistic where lighter and darker colors indicate higher or lower shared genetic affinities, respectively.

We then tested the similarity of Bela III genome to modern-day West-Eurasian populations using *f*_*3*_-outgroup statistics^49^, which measure shared genetic affinity compared to a common outgroup, here the African Mbuti population. Consistent with the PCA result, the genome of Bela III clusters within the diversity of present-day Europeans and indistinguishable from most other European populations (Fig. 2). In order to assess any differential similarity of other present-day populations to Bela III compared to Croatians and Hungarians we tested *f*_*4*_-statistic^49^ in the form *f*_*4*_*(X, Mbuti, Croatians/Hungarians, Bela III)*, where *X* is a list of current worldwide populations (Table S7). None of the tested comparisons reported significant deviation from zero (Z score above -3) confirming that most of the King’s genetic ancestry is shared with present-day Croatians and Hungarians. However, tests invoking East Eurasian and Oceanian populations, such as Papuan, Ami and Han, resulted in a marginally significant attraction to Bela III (Z score around 2). This suggests a slightly higher allele sharing between the King’s genome and present-day Asians than to those European populations that fall in closest proximity in PCA space.

### Phenotype analysis

We finally inspected phenotypic related SNPs to infer features of the physical appearance and metabolism of Bela III (Table S8). As reported in historical representations, the King likely had light skin and blue or green eyes because of the presence of derived alleles at defined loci on the SLC45A2, SLC24A5 and HERC2 genes. Moreover, he was likely to be lactose tolerant, showing the derived variant on the LCT gene (rs4988235), while he carried the ancestral variant of the hair thickness-related EDAR gene, like most Europeans today.

## Discussion

A few decades after the collapse of the Avar Khaganate (ca. 822 AD), Álmos and his son Árpád conquered the Carpathian Basin (ca. 862–895 AD)^28^. Three of the steppe empires based in the Carpathian Basin provided an alternative to the post-Roman model of government^50^. During the conquest, Hungarian invaders, together with Turkic-speaking Kabars assimilated the Avars and Slavonic groups^29^. Moreover, it is suggested that Hungarian conquerers together with the Turkic-speaking Kabars moved in and integrated the “Avar” (including Onoghurs, Proto-Hungarians etc.) people.

Genetic studies of mtDNA, Y chromosomal and autosomal markers from those conquering Hungarians have revealed an admixed ancestry characterized by both western and eastern Eurasian genetic components^20–22^. However, multiple early Medieval human migrations took place after the collapse of the Roman Empire in Hungary. Therefore, the reconstruction of the population dynamics that took place in the region until the establishment of the Árpád dynasty would require archaeogenetic data from this intermediate period. Indeed, mtDNA and Y chromosomal haplogroups of Hun- and Avar-related individuals from Hungary suggest an even stronger Eastern Eurasian genetic influence than in groups from the Hungarian conquest-period^20,23,24^.

Our study presents the first genome-wide analysis of a historical Hungarian individual older than the sixth century AD, by directly investigating the genomic profile of one of the most prominent kings of the Árpád dynasty. Historical evidence reveals that from the initial conquest of the Carpathian Basin by the Hungarian tribal federation in 895 to King Béla III (1172–1196) a paternal line of inheritance was always followed (Figure S1). In 1170 Béla III married Anna of Antioch (daughter of Raynald Châtillon) following the common tradition of combined marriages with members of other European noble families to create or maintain strategic diplomatic relationships. We reconstructed genome-wide data from two anatomical elements of Bela III with over 1.2 million SNPs covered at an average depth of six-fold.

The autosomal DNA profile of Bela III falls within the genetic variation of present-day east European populations like Croatians and Hungarians. This is further supported through his mtDNA genome that belongs to hg H, the most common European maternal lineage. Furthermore, he was previously found to carry Y-chromosome hg R1a that can be traced back to Central Asia based on a more detailed phylogeographic reconstruction^33^. Based on the established genealogy (Figure S1), this Y-chromosome lineage provides a direct link between Bela III and his ancestor Árpád (845–907 AD), the Great Prince of the Hungarians who started the so-called Árpád Dynasty, if there was no hidden paternity present during the line. It is therefore possible that Great Prince Árpád and later family members had much larger proportions of Eastern Eurasian ancestry than Bela III. However, this could have been lost in Bela III because of repeated marriages with members of noble families of European descent during the 18 generations (~ 300 years) that separated the two Hungarian rulers.

Nevertheless, the paternal relationship between Bela III and Álmos may extend the presence of this Eastern Eurasian Y-chromosomal haplogroup in Hungary back to at least the end of the ninth century AD. Such eastern Eurasian-related Y-chromosomes could, however, been also acquired locally, since three elite military individuals from the Hun and the Early Avar period have been recently found belonging to the same overarching hg R1a1a1b2-Z93^23^. Alternatively, this eastern Eurasian legacy could have arrived in Hungary with the conquering Hungarian tribes through additional population movements from the East. Of note, the genetic composition of the local population at that time may have differed from the one observed in members of the ruling dynasty. Therefore, since the conquering tribes are considered responsible for bringing the current language into Hungary it would be important to genetically characterize the genome of even earlier representatives of the Hungarian elite. This would determine whether the eastern Eurasian ancestry reported in the Y-chromosome of King Bela III was acquired locally or arrived through additional east to west migrations and also test if Great Prince Árpád and later family members had much larger proportions of Eastern Eurasian autosomal ancestry than the later Bela III.

## Methods

### Sampling strategy

We collected four bone fragments from the tomb associated with King Bela III. His remains were buried in the church (Székesfehérvár) and in 1848 they were transported to Budapest and nowadays the sarcophagus is in the Mathias Church^51^. The tomb of Béla III and Anna was opened in 21–22.03.2014. The boxes with the skeleton were transported from the church. The bone specimens were collected at the National Institute of Oncology under sterile conditions. Samples were taken from the skeletons which were stored before and during the sampling at room temperature. Approximately 2–5 g pieces of bones (femur, vertebra, metatarsal and tarsal) and were put in sterile Falcon tubes and were immediately frozen in dry ice. Bones in one of the tubes were divided into two approximately equal pieces. They were transferred in May of 2014 to the laboratories of Suzanne Hummel and Johannes Krause. P.I. Nagy was invited to the project in October 2014 by BM. He received DNA isolated from all bones in Hummel’s laboratory in 2015 via BM, he reported his first sequence reports in August 2015. We showed the sample details in Table S9. We presented the preliminary results including the full and complete Y chromosome haplotype at the consortium meeting at the National Institute of Oncology in Budapest on September 1, 2015 (BM presentation).

### Laboratory procedures

The ancient DNA workflow for this study was carried out at the Laboratory of Archaeo- and Paleogenetics at the University of Tübingen. Four skeletal elements associated to King Bela III (femur, vertebra, metatarsal and tarsal bones) were collected from the royal graves discovered in 1848 in the Royal Basilica of Székesfehérvár in Budapest (Hungary). All four specimens were sampled using a dentist drill (Nakanishi (Emax Evolution)) and the resulting bone powder was used to extract DNA following an established protocol^35^. Sequencing libraries were initially built with a double-stranded protocol without DNA damage correction^36^ and then indexed with double barcodes^52^. After amplification, libraries were diluted equimolarly and shotgun sequenced on Illumina platforms (HiSeq4000, NextSeq500) in paired-end mode. Two out of four libraries form Bela III with highest percentage of aDNA (MA32 and MA33) were enriched for the complete mitochondrial genome using DNA capture^53^ and then paired-end sequenced on Illumina NextSeq500 machine. Furthermore, from two DNA extracts of Bela III single-stranded libraries were generated (MA172 and MA173) following a protocol that retains C to T damage-caused by substitutions in the last two terminal positions from both ends^37,54^. Subsequently an in-solution nuclear DNA capture was used targeting either ~ 390,000 or ~ 1,240,000 SNPs scattered across the human genome^6,38^ for the two Bela III double stranded libraries (MA32 and MA33). The enriched libraries were paired-end sequenced on Illumina NextSeq500 machine. DNA sequences from shotgun sequencing screening, nuclear and mtDNA capture were processed through EAGER^55^. Adapters were trimmed, forward and reverse reads were merged and fragments below 30 bp long were removed^55^. The resulting sequences were aligned against the mtDNA and nuclear DNA reference genomes (rCRS and hg19, respectively) followed by mapping quality filtering (q30) and duplicate removal.

### Sex determination

The sex of the newly reported samples in this study was determined by counting the number of reads overlapping with the targets of 1240 k capture reagent as discussed in Fu et al.^38^. Reads of high base and mapping quality (SAMtools depth -q30 -Q37) were extracted using SAMtools v1.3.1^56^. Ratios of the numbers of reads mapped on X chromosome or Y chromosome were calculated and compared with that mapped on autosomes (X-rate = xCov/autCov and Y-rate = yCov/autCov).

### mtDNA contamination and haplogroup assignment

MtDNA contamination level and reconstructed mtDNA consensus sequence of both individuals were estimated using schmutzi^43^. Haplogroup assignment was performed with the online tool Haplofind^57^.

### Nuclear contamination Estimation

An X-chromosomal contamination test was performed for the five libraries of Bela III following an approach introduced by Rasmussen et al. and implemented in the ANGSD software suite^58^. The Method of Moments (MOM) and Maximum Likelihood (ML) estimate from “Method 1” and “Method 2” likelihood computation were applied using this software package. A consistent estimate of low contamination rate (about 1.2%-2.7%) in different libraries using different methods was determined.

### SNP calling

The sequence data was demultiplexed, adaptor clipped with leehom and then further processed using EAGER^53^, which included mapping with BWA (v0.6.1)^59^ against human genome reference GRCh37/hg19, and removing duplicate reads with the same orientation and start and end positions. To avoid an excess of remaining C-to-T and G-to-A transitions at the ends of the reads, three bases of the ends of each read were clipped for each sample. “Pseudo-haploid” calls were generated by selecting a single read randomly at each of the targeted 1240 k SNP positions.

### Populations for comparisons

The genomic data of populations used for comparisons are from the Human Origin Dataset: https://reich.hms.harvard.edu/allen-ancient-dna-resource-aadr-downloadable-genotypes-present-day-and-ancient-dna-data.

### PCA projection

Principal component analysis was carried out in the smartpca program of EIGENSOFT^47^, using default parameters and the lsqproject: YES and numoutlieriter: 0 options. We project our ancient Bela III sample onto the variation of present-day West Eurasians from published Human Origin Dataset over 591,642 SNPs. We have not used the option autoshrink: YES or shrinkmode: YES.

### ADMIXTURE

We carried out ADMIXTURE^48^ analysis after pruning for linkage disequilibrium in PLINK^60^ with parameters –indep-pairwise 200 25 0.4 which retained 252,493 SNPs. We ran ADMIXTURE with default fivefold cross-validation (–cv = 5), varying the number of ancestral populations between K = 2 and K = 12 in 100 bootstraps with different random seeds. The samples used in this analysis are present-day West Eurasians, and individuals from worldwide representative populations Mbuti, Yoruba, Han, Papuan, Karitiana, Eskimo, Uzbek, Ami, Selkup, and Kalash. We observed the lowest CV errors for K = 7.

### Pairwise outgroup *f*_*3*_ statistics

Outgroup *f*_*3*_-statistics of the form *f*_*3*_ (Mbuti; X, Y) were used in order to test which West Eurasian populations share the most genetic drift with Bela III. Analysis of *f*_*3*_-statistics was carried out using ADMIXTOOLS^49^ with standard errors computed with a block jackknife.

### *f*_*4*_ statistics

Outgroup *f*_*4*_-statistics of the form (Worldwide populations, Mbuti; BelaIII, Hungarian/Croatian) were used to test if Africans, Europeans, South Asians, East Asians, Native Americans and Oceanians share extra affinity with Bela III than with present-day Hungarian and Croatian. Analysis of *f*_*4*_-statistics was carried out using ADMIXTOOLS with standard errors computed with a block jackknife. The statistics are consistent with 0, implying there was no extra ancestry from outside Europe in Bela III.

### Y chromosomal haplogroup analysis

The Y chromosomal haplogroup was determined by examining a set of diagnostic positions on chromosome Y using the ISOGG (http://isogg.org/) and also Y-full (https://www.yfull.com/tree/) database. To perform this analysis, we restricted our analysis to only include reads with a mapping quality higher than 30. Afterwards, we determined the haplogroups by identifying the most derived Y chromosomal SNP in our individual. The sample has a derived allele at R1a1a1b2a2a-Z2123: C → T with coverage of 3x. Upstream mutations assigning our individual to R1a1a1b2a-Z95 and R1a1a1b2-M746 could be identified.

### Phenotype analysis

#### Allele information of SNPs thought to be affected by selection in samples

Only high-quality (q > 30) bases are counted. rs4988235 is responsible for lactase persistence in Europe. The SNPs at *SLC24A5* and *SLC45A2* are responsible for light skin pigmentation. The SNP at *EDAR* affects tooth morphology and hair thickness. The SNP at *HERC2* is the primary determinant of light eye color in present-day Europeans.

## Supplementary Information

Supplementary Information.

Supplementary Information.

## Data Availability

The datasets generated during and analysed during the current study are available in Zenodo, at https://zenodo.org/record/6367404#.YkRbTajTXIV.
