# Author Correction: Genome-wide autosomal, mtDNA, and Y chromosome analysis of King Bela III of the Hungarian Arpad dynasty

**DOI:** 10.1038/s41598-022-11779-4

**Published:** 2022-05-03

**Authors:** Chuan‑Chao Wang, Cosimo Posth, Anja Furtwängler, Katalin Sümegi, Zsolt Bánfai, Miklós Kásler, Johannes Krause, Béla Melegh

**Affiliations:** 1 Department of Archaeogenetics, Max Planck Institute for the Science of Human History, 07745 Jena, Germany; 2 Department of Anthropology and Ethnology, Institute of Anthropology, School of Sociology and Anthropology, State Key Laboratory of Cellular Stress Biology, State Key Laboratory of Marine Environmental Science, National Institute for Data Science in Health and Medicine, School of Life Sciences, Xiamen University, Xiamen, 361005 China; 3 Institute for Archaeological Sciences, Archaeo- and Palaeogenetics, University of Tübingen, 72070 Tübingen, Germany; 4 Department of Medical Genetics, Medical School, University of Pécs, Szigeti u. 12, Pécs, 7624 Hungary; 5 Szentágothai Research Center, University of Pécs, Ifjúság út 24, Pécs, 7624 Hungary; 6 Department of Biochemistry and Medical Chemistry, Medical School, University of Pécs, Szigeti u. 12, Pécs, 7624 Hungary; 7 National Institute of Oncology, Rácz Gy. u. 7‑9, Budapest, 1122 Hungary

Correction to: *Scientific Reports* https://doi.org/10.1038/s41598-021-98796-x, published online 28 September 2021

The Data Availability section in the original version of this Article was omitted. The Data Availability section now reads:

Data Availability:

The datasets generated during and analysed during the current study are available in Zenodo, at https://zenodo.org/record/6367404#.YkRbTajTXIV.

The original Article has been corrected.

