# Supplementary material for: Genome-wide autosomal, mtDNA, and Y chromosome analysis of King Bela III of the Hungarian Arpad dynasty
Source: Sci Rep. 2021 Sep 28;11:19210. doi: 10.1038/s41598-021-98796-x (PMC8478946; doi:10.1038/s41598-021-98796-x)
Supplement: Supplementary file 1 — Supplementary Information. [file 41598_2021_98796_MOESM1_ESM.pdf]

Genome-wide autosomal, mtDNA, and Y chromosome analysis of King Bela III of the Hungarian Arpad dynasty

Chuan-Chao Wang, Cosimo Posth, Anja Furtwängler, Katalin Sümegi, Zsolt Bánfai, Miklós Kásler, Johannes Krause, Béla Melegh

Supplementary Material

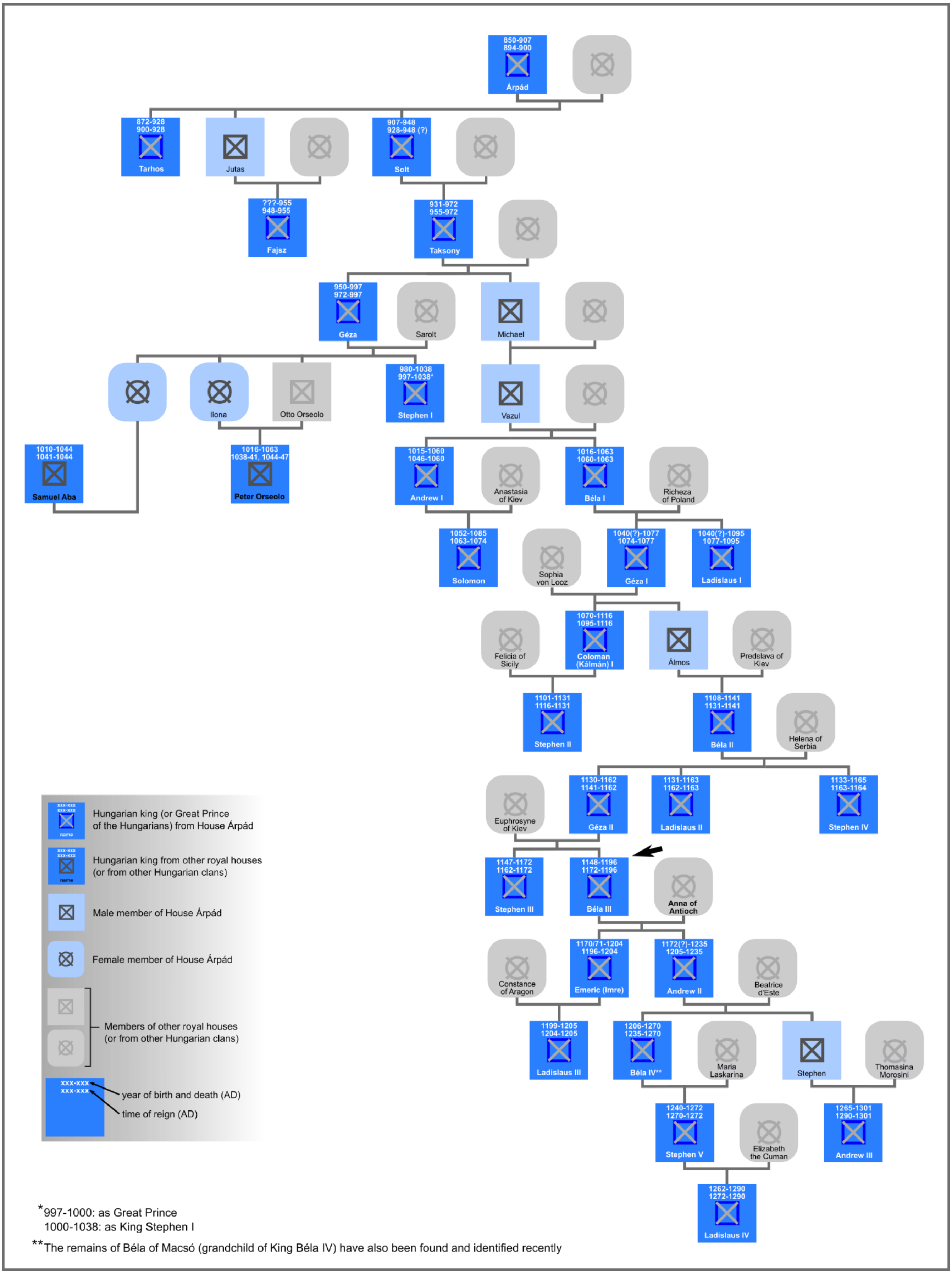

Figure S1. Reconstructed genealogy of the Árpád dynasty based on written evidences. King Bela III placement along the male lineage is indicated with an arrow.

Based on: Glatz, F. A magyarok krónikája. [The Chronicle of the Hungarians] (Helikon, Pécs, 2006). ISBN: 963-227-070-3.

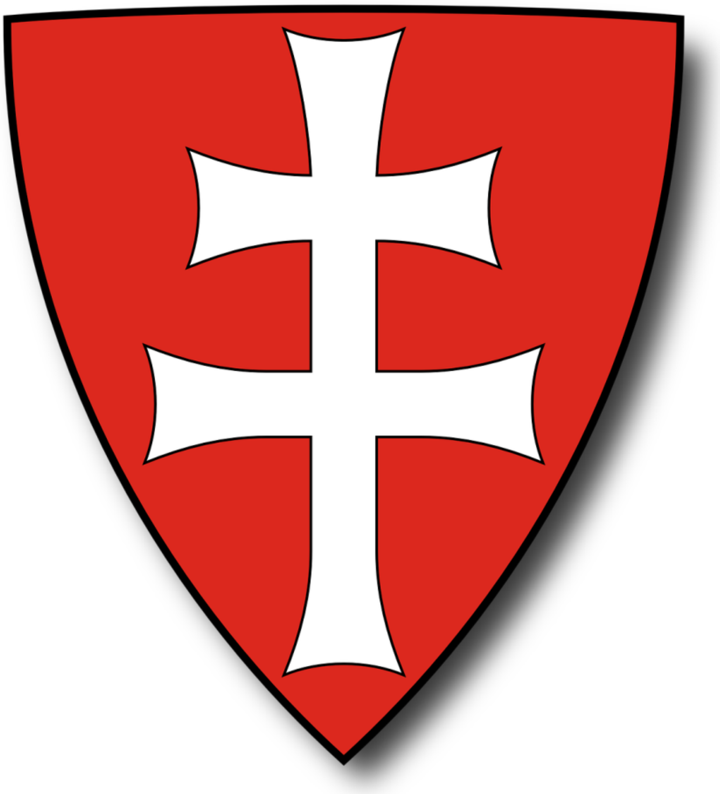

Figure S2. The “double-cross” as the symbol of the supreme was introduced by King Béla III. It has been put on coat of arms by his grandson King Béla IV, thus the silver “double-cross” in the red field the became a heraldic symbol. Image credits: public domain, CC0.

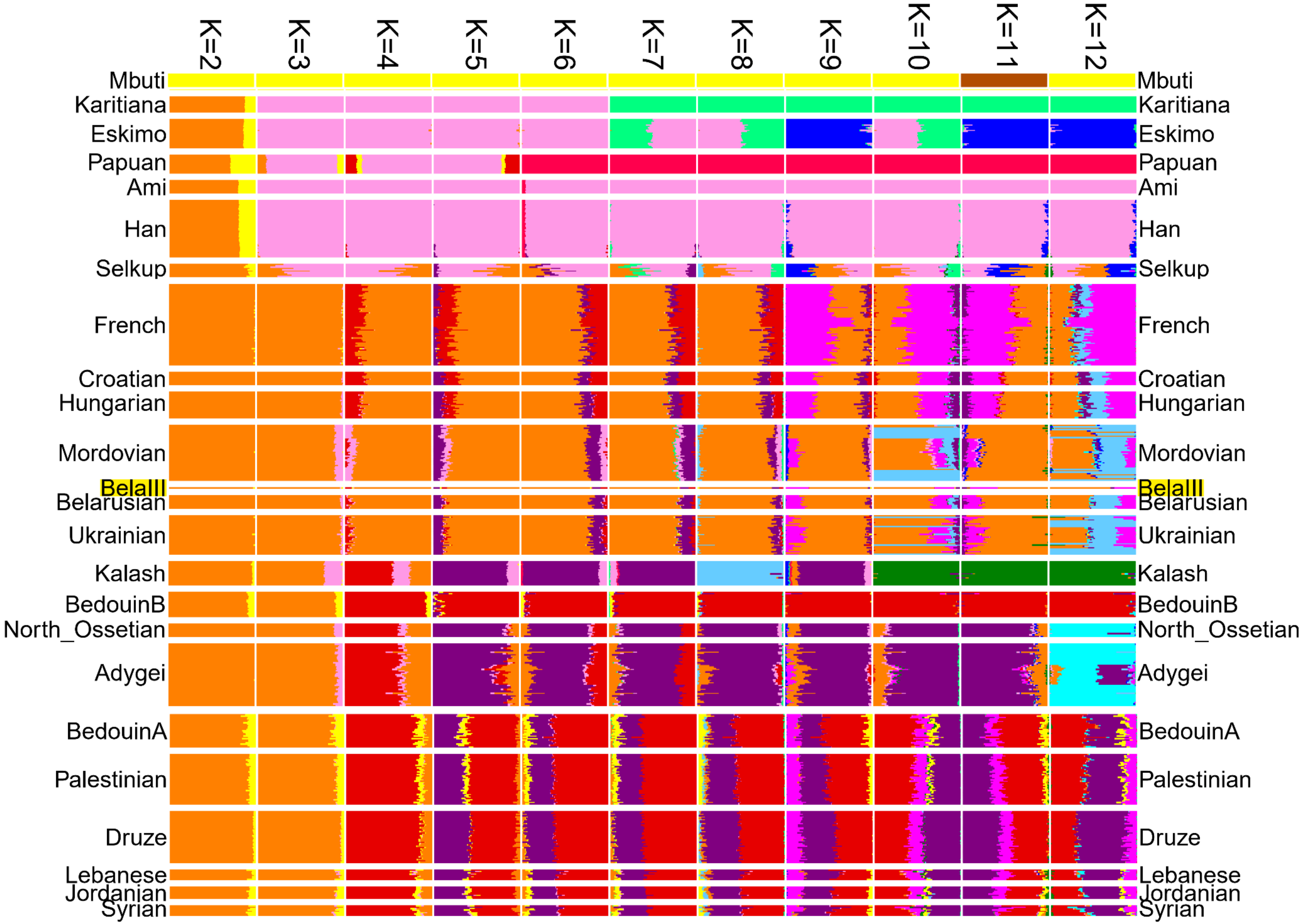

Figure S3. ADMIXTURE analyses reported applying K=2 to K=12 hypothetical ancestral groups.

Table S1. Extraction and library names

Lab ID	Extract	Shotung (ds_noUDG)	mtDNA captured (ds_noUDG)	390K captured (ds_noUDG)	1240K captured (ss_halfUDG)
JK2069	MX27	MA30s	-	-	-
JK2070	MX28	MA31s	-	-	-
JK2071	MX29	MA32s	MA32m	MA32snp	MA172snp
JK2072	MX30	MA33s	MA33m	MA33snp	MA173snp

Table S2. Shotgun, mtDNA capture and nuclear capture summary statistics.

Shotgun

Sample Name	# of Raw Reads prior Clip & Merge (C&M)	# reads after C&M prior mapping	Mapped Reads after RMDup	Cluster Factor	Endogenous DNA (%)	# of reads on mitochondrium	MT/NUC Ratio	DMG 1st Base 3'	DMG 1st Base 5'	Average fragment length
MA30s	4497148	2459777	27839	1.005	1.673	17	84.41	0.1613	0.1625	71.52
MA31s	4079186	2029951	30813	1.005	1.907	283	1717.13	0.1122	0.1093	69.16
MA32s	3943628	2060190	137712	1.005	9.002	104	146.91	0.1382	0.1415	74.49
MA33s	3932648	2033203	119898	1.006	7.683	82	116.91	0.1118	0.1141	80.72

mtDNA capture

Sample Name	# of Raw Reads prior Clip & Merge (C&M)	# reads after C&M prior mapping	# of Merged Reads	Mapped Reads after RMDup	Cluster Factor	Endogenous DNA (%)	Mean Coverage	Coverage >= 1X in %	DMG 1st Base 3'	DMG 1st Base 5'	Average fragment length
MA32m	949420	482101	460694	64593	1.297	18.189	351.61	100	0.156	0.1606	90.19
MA33m	901614	458488	434503	52698	1.209	14.662	316.5832	100	0.1169	0.1235	99.54

Nuclear capture

Library ID	Sample	Library protocol	# of Raw Reads prior Clip & Merge (C&M)	# reads after C&M prior mapping	Mapped Reads after RMDup	Cluster Factor	Endogenous DNA (%)	DMG 1st Base 3'	DMG 1st Base 5'	Average fragment length
MA172	BelaIII	ss_halfUDG	35468548	35468548	6666586	2.172	40.824	0.0559	0.0768	55.9
MA173	BelaIII	ss_halfUDG	30870712	30870712	5543457	2.069	37.159	0.0502	0.0586	62.79
MA32	BelaIII	ds_noUDG	27505036	27505036	3999450	1.06	15.409	0.1199	0.1362	73.3
MA33	BelaIII	ds_noUDG	35678385	35678385	4656410	1.066	13.912	0.0897	0.1002	80.06

Table S3. genetic sex determination.

Sample	Y-rate	X-rate	sex
BelaIII	0.524564	0.426962	Male

Table S4. X-chromosome contamination estimate for each library.

Library	Number of SNPs	Methods	version	MoM	SE(MoM)	ML	SE(ML)
MA172	4251	Method1	old_llh	0.021765	2.53E-03	0.027055	0.00E+00
		Method1	new_llh	0.021706	2.53E-03	0.026995	1.11E-13
		Method2	old_llh	0.022315	4.16E-03	0.023601	9.02E-14
		Method2	new_llh	0.022255	4.16E-03	0.023561	9.32E-14
MA173	4101	Method1	old_llh	0.017561	2.30E-03	0.021908	3.15E-14
		Method1	new_llh	0.017519	2.31E-03	0.021865	9.22E-14
		Method2	old_llh	0.012945	3.45E-03	0.013916	6.31E-14
		Method2	new_llh	0.012915	3.46E-03	0.013896	4.32E-14
MA32	2561	Method1	old_llh	0.01291	3.14E-03	0.015401	5.14E-14
		Method1	new_llh	0.012851	3.16E-03	0.015355	1.31E-14
		Method2	old_llh	0.010714	4.69E-03	0.011181	3.67E-14
		Method2	new_llh	0.010665	4.70E-03	0.011174	1.11E-14
MA33	3174	Method1	old_llh	0.01488	2.65E-03	0.018411	5.16E-14
		Method1	new_llh	0.014835	2.66E-03	0.01837	7.02E-14
		Method2	old_llh	0.024161	5.09E-03	0.024423	1.21E-14
		Method2	new_llh	0.024087	5.10E-03	0.024387	4.42E-14

Table S5. mtDNA contamination estimates and mtDNA haplogroups

Sample Name	Contamination (Low-High)	MtDNA haplogroup
MA32m	0.01 (0-0.02)	H1b
MA33m	0.01 (0-0.02)	H1b

Table S6. Y-chromosome derived SNPs defining the assigned hg.

SNP	Haplogroup	rs number	Ypos37	ancestral	derived	BelaIII	depth
PF6014	R	rs1865680	6868118	A	G	A	2
P224	R	rs17307398	17285993	C	T	T	4
M734	R	rs1348733	18066156	C	T	T	9
P285	R	rs17249974	19267344	C	A	A	2
P227	R	rs4481791	21409706	G	C	C	3
P280	R	rs891407	21843090	C	G	G	1
P294	R1	rs1005041	7570822	G	C	C	4
P242	R1	rs7067478	7647357	G	A	A	2
P238	R1	rs9785717	7771131	G	A	A	4
P231	R1	rs9786465	9989615	A	G	G	2
P225	R1	rs17307070	15590342	G	T	T	1
P286	R1	rs1118473	17716251	C	T	T	3
P236	R1	rs9785959	17782178	C	G	G	10
P233	R1	rs9786232	21166358	T	G	G	2
M306	R1	rs1558843	22750583	C	A	A	2
L145	R1a	rs17306692	14138745	C	A	A	4
L62	R1a	rs17222573	17891241	A	G	G	16
L146	R1a	rs17250535	23473201	T	A	A	2
Page65.2	R1a1	rs2534636	2657176	C	T	T	2
M459	R1a1	rs17316227	6906074	A	G	G	7
M515	R1a1a	rs17221601	14054623	T	A	A	3
L168	R1a1a		16202177	A	G	A	1
M512	R1a1a	rs17222146	16315153	C	T	T	2
M514	R1a1a	rs17315926	19375294	C	T	T	1
L449	R1a1a		22966756	C	T	T	6
M417	R1a1a1	rs17316771	8533735	G	A	A	1
Page7	R1a1a1	rs34297606	14498990	C	T	T	1
S441	R1a1a1b	rs112284571	7683058	G	A	A	1
S224	R1a1a1b	rs111731595	8245045	C	T	T	1
M746	R1a1a1b2		18985344	C	A	A	5
Z95	R1a1a1b2a		23956870	C	T	T	3
Z2123	R1a1a1b2a2a		16453077	C	T	T	3

Table S7. f_4-statistics results.

Worldwide	Outgroup	BelaIII	Hungarian/Croatian	f_4	Z	SNPs
Yoruba	Mbuti	BelaIII	Croatian	0.0004	0.229	543701
Han	Mbuti	BelaIII	Croatian	0.0056	1.872	543701
Papuan	Mbuti	BelaIII	Croatian	0.0072	2.194	543701
Karitiana	Mbuti	BelaIII	Croatian	0.0031	0.814	543701
Eskimo	Mbuti	BelaIII	Croatian	0.0047	1.445	543701
Uzbek	Mbuti	BelaIII	Croatian	0.0022	0.891	543701
Ami	Mbuti	BelaIII	Croatian	0.0064	1.989	543701
Selkup	Mbuti	BelaIII	Croatian	0.0051	1.78	543701
Kalash	Mbuti	BelaIII	Croatian	0.0032	1.176	543701
Iranian	Mbuti	BelaIII	Croatian	0.0009	0.383	543701
BedouinB	Mbuti	BelaIII	Croatian	0.0003	0.124	543701
Sardinian	Mbuti	BelaIII	Croatian	0.0004	0.154	543701
Adygei	Mbuti	BelaIII	Croatian	0.002	0.807	543701
Russian	Mbuti	BelaIII	Croatian	0.0023	0.893	543701
Druze	Mbuti	BelaIII	Croatian	0.0001	0.045	543701
Yoruba	Mbuti	BelaIII	Hungarian	0.0015	0.863	543701
Han	Mbuti	BelaIII	Hungarian	0.0038	1.264	543701
Papuan	Mbuti	BelaIII	Hungarian	0.0066	2.037	543701
Karitiana	Mbuti	BelaIII	Hungarian	0.001	0.262	543701
Eskimo	Mbuti	BelaIII	Hungarian	0.0032	1.003	543701
Uzbek	Mbuti	BelaIII	Hungarian	0.0015	0.595	543701
Ami	Mbuti	BelaIII	Hungarian	0.0046	1.431	543701
Selkup	Mbuti	BelaIII	Hungarian	0.0036	1.265	543701
Kalash	Mbuti	BelaIII	Hungarian	0.0029	1.099	543701
Iranian	Mbuti	BelaIII	Hungarian	0.0023	0.962	543701
BedouinB	Mbuti	BelaIII	Hungarian	0.0026	1.035	543701
Sardinian	Mbuti	BelaIII	Hungarian	0.0017	0.666	543701
Adygei	Mbuti	BelaIII	Hungarian	0.0028	1.131	543701
Russian	Mbuti	BelaIII	Hungarian	0.0019	0.758	543701
Druze	Mbuti	BelaIII	Hungarian	0.0021	0.851	543701

Table S8. Number of independent DNA fragments overlapping five phenotype-related SNPs with the frequency of the respective derived allele.

		LCT	SLC45A2	SLC24A5	EDAR	HERC2
	SNP	rs4988235	rs16891982	rs1426654	rs3827760	rs12913832
	Ancestral	G	C	G	A	A
	Derived	A	G	A	G	G
Bela III	Coverage	8	24	5	11	7
	Derived	75%	100%	100%	0%	28.6%

Table S9. The sample details of laboratory procedures.

Anatomical Element	Lab ID	amount of bone powder (mg)	Extract	Shotgun library (ds_noUDG)	mtDNA captured library (ds_noUDG)	390K captured library (ds_noUDG)	1240K captured library (ss_halfUDG)
Femur	JK2069	111	MX27	MA30s	-	-	-
Vertebra	JK2070	92	MX28	MA31s	-	-	-
Metatarsal	JK2071	107	MX29	MA32s	MA32m	MA32snp	MA172snp
Tarsal	JK2072	97	MX30	MA33s	MA33m	MA33snp	MA173snp
