# Supplementary material for: Genome-wide autosomal, mtDNA, and Y chromosome analysis of King Bela III of the Hungarian Arpad dynasty
Source: Sci Rep. 2021 Sep 28;11:19210. doi: 10.1038/s41598-021-98796-x (PMC8478946; doi:10.1038/s41598-021-98796-x)
Supplement: Supplementary file 2 — Supplementary Information. [file 41598_2021_98796_MOESM2_ESM.docx]

# Supplementary Material

**Figure S1.** Reconstructed genealogy of the Árpád dynasty based on written evidences. King Bela III placement along the male lineage is indicated with an arrow.

Based on: Glatz, F. A magyarok krónikája. [The Chronicle of the Hungarians] (Helikon, Pécs, 2006). ISBN: 963-227-070-3.

**Figure S2.** The “double-cross” as the symbol of the supreme was introduced by King Béla III. It has been put on coat of arms by his grandson King Béla IV, thus the silver “double-cross” in the red field the became a heraldic symbol. Image credits: public domain, CC0.

**Figure S3.** ADMIXTURE analyses reported applying K=2 to K=12 hypothetical ancestral groups.

**Table S1.** Extraction and library names

**Table S2.** Shotgun, mtDNA capture and nuclear capture summary statistics.

**Table S3.** Genetic sex determination.

**Table S4.** X-chromosome contamination estimate for each library.

**Table S5.** mtDNA contamination estimates and mtDNA haplogroups

**Table S6.** Y-chromosome derived SNPs defining the assigned hg (Ypos37 refers to “Y-chromosome position on Genome Reference Consortium Human Build 37”).

**Table S7.** *f_4_*-statistics results.

**Table S8.** Number of independent DNA fragments overlapping five phenotype-related SNPs with the frequency of the respective derived allele.

**Table S9**. The sample details of laboratory procedures.
